# Whole exome sequencing identifies a novel mutation in *ASPM* and ultra-rare mutation in *CDK5RAP2* causing Primary microcephaly in consanguineous Pakistani families

**DOI:** 10.12669/pjms.38.1.4464

**Published:** 2022

**Authors:** Ehtisham ul Haq Makhdoom, Haseeb Anwar, Shahid Mahmood Baig, Ghulam Hussain

**Affiliations:** 1 Ehtisham ul Haq Makhdoom (MPhil), Neurochemicalbiology and Genetics Laboratory (NGL), Department of Physiology, Faculty of Life Sciences, Government College University, 38000, Faisalabad, Pakistan. Human Molecular Genetics Laboratory, Health Biotechnology Division, NIBGE College, PIEAS, 38000, Faisalabad, Pakistan; 2 Haseeb Anwar (PhD), Neurochemicalbiology and Genetics Laboratory (NGL), Department of Physiology, Faculty of Life Sciences, Government College University, 38000, Faisalabad, Pakistan; 3 Shahid Mahmood Baig (PhD), Department of Biological and Biomedical Sciences, The Aga Khan University, 74000, Karachi, Pakistan. Pakistan Science Foundation, Constitution Avenue, 44000, Islamabad, Pakistan. Human Molecular Genetics Laboratory, Health Biotechnology Division, NIBGE College, PIEAS, 38000, Faisalabad, Pakistan; 4 Ghulam Hussain (PhD), Neurochemicalbiology and Genetics Laboratory (NGL), Department of Physiology, Faculty of Life Sciences, Government College University, 38000, Faisalabad, Pakistan

**Keywords:** *ASPM*, *CDK5RAP2*, Novel mutation, Pakistani, Primary Microcephaly, Whole-exome sequencing

## Abstract

**Background & Objectives:**

Primary Microcephaly (MCPH) is a rare neurogenetic disease, manifesting congenitally reduced head circumference and non-progressive intellectual disability (ID). To date, twenty-eight genes with biallelic mutations have been reported for this disorder. The study aimed for molecular genetic characterization of Pakistani families segregating MCPH.

**Methods:**

We studied two unrelated consanguineous families (family A and B) presenting >2 patients with diagnostic symptoms of MCPH, born to asymptomatic parents. We employed whole-exome sequencing (WES) of probands to find putative causal mutations. The candidate variants were further confirmed and analyzed for co-segregation by Sanger sequencing of all available members of each family. This study was conducted at Government College University, Faisalabad, Pakistan, and Cologne Center for Genomics (CCG), University of Cologne, Germany; during 2017-2020.

**Results:**

We identified a novel homozygous variant c.10097_10098delGA, p.(Gly3366Glufs*19) in exon 26 of *ASPM* gene in family A which presents with moderate intellectual disability, speech impairment, visual abnormalities, seizures, and ptyalism. Family B was found to segregate nonsense, homozygous variant c.448C>T p.(Arg150*) in *CDK5RAP2*. The patients also exhibited mild to severe seizures without ptyalism that has not been previously reported in patients with mutations in the *CDK5RAP2* gene.

**Conclusion:**

We report a novel mutation in *ASPM* and ultra-rare mutation in the *CDK5RAP2* gene, both causing primary microcephaly. The study expands the mutational spectrum of the *ASPM* gene to 212, and also adds to the clinical spectrum of *CDK5RAP2* mutations. It also demonstrated the utility of WES in the investigation and genetic diagnosis of genetically heterogeneous disorders like MCPH. These findings would aid in diagnostic and preventive strategies including carrier screening, cascade testing, and genetic counselling.

## INTRODUCTION

Autosomal recessive primary microcephaly (MCPH, MIM251200) refers to congenitally reduced occipitofrontal circumference/ head circumference (HC) by >3 standard deviation (SD) as compared to mean for same age and gender along with sloping forehead and non-progressive cognitive impairment. It is the manifestation of insufficient growth of the foetal brain, mainly the cerebral cortex, during the antenatal period.^1^ Cerebral cortex is the most intricate part of the human brain and it normally contains about 10 neuronal cells, with more than 10 connections that control learning, memory, and cognition, etc.^2^ MCPH patients have decreased volume of the cerebral cortex and simplified gyration with overall normal morphology of the brain.^1^ The estimated global incidence of MCPH is 1.3-150/ 100,000 live births^3^ rising to as high as one in 10,000 in the inbred population of Pakistan.^4^

MCPH is a genetically heterogeneous disease with autosomal recessive inheritance. So far, 26 genes have been reported for this disorder that include, *Microcephalin*, *WDR62*, *CDK5RAP2*, *CASC5*, *ASPM*, *CENPJ*, *STIL*, *CEP135*, *CEP152*, *ZNF335*, *PHC1*, *CDK6*, *CENPE*, *SASS6*, *MFSD2A*, *ANKLE2*, *CIT*, *WDFY3, COPB2*, *KIF14* *NCAPD2*, *NCAPD3*, *NCAPH*, *NUP37, RRP7A* and recently, *MAP11*^4^ Products of these genes function in diverse cellular processes critical for optimal neurogenesis such as DNA repair, cell cycle regulation, centrosome maturation, position, duplication, spindle orientation, and positioning.^5^ Neurogenesis in the cerebral cortex is derived by rounds of symmetrical (proliferative) or asymmetrical (differentiating) divisions in neuro-progenitor cells. During symmetric division, the mitotic spindle is oriented in the plane of neuro-epithelium (cortical plane) resulting in two neuro-progenitor cells. During asymmetric division, the mitotic spindle lies perpendicular to the cortical plane yielding a single neuro-progenitor cell and a terminally differentiated neuron. Mutations in MCPH genes cause dysregulation of these processes, leading to premature depletion of neuro-progenitor cells and subsequent reduction in the final neuronal count of the cerebral cortex.^6^ Disrupted function of some of these genes causes enhanced apoptosis in of the progenitor cells, which leads to a reduced number of glial and neuronal cells, leading to MCPH phenotype.^7^

Out of 25 MCPH genes, *ASPM* (Abnormal Spindle-like Microcephaly-Associated, MIM#605481) has been reported most frequently, accounting for about 68% of cases globally.^4^ Locating at 1q31.3, the 62,567 base pairs (bp) long gene spans over 28 exons with the open reading frame of 10,906 bps, coding for 3,477 amino acids long ASPM protein.^8^ ASPM localizes at the pericentrosomal region at mitotic spindle poles; midbody at cytokinesis; cytoplasmic at interphase and is known to mediate orientation and organization of spindle poles.^9^

*CDK5RAP2* (cyclin dependant kinase 5 regulatory associated protein 2, MIM#608201) is a relatively rarely reported gene for MCPH. At chromosomal position 9q33.2, *CDK5RAP2* gene comprises 191,290 bps, split into 38 exons coding for 1893 amino acids long protein.^3^ The protein is expressed at the centrosome throughout the cell cycle and at the midbody during cytokinesis. It mediates maintenance of microtubule dynamics, recruitment and stabilization of pericentriolar matrix, centrosome maturation, and cohesion.^10^

In this study, we report clinical and genetic findings of two Pakistani families segregating primary microcephaly, carrying a novel homozygous variant in *ASPM* c.10097_10098delGA, p.(Gly3366Glufs*19) and an already reported c.448C>T, p.(Arg150*) variant in *CDK5RAP2*.

## METHODS

### Samples collection:

The study was conducted at Government College University Faisalabad (GCUF) Pakistan and Cologne Center for Genomics (CCG), University of Cologne, Germany from 2017 to 2020. It was approved by the Institutional Review Board (IRB-3826) of GCUF. In this study, two unrelated consanguineous families with multiple affected individuals segregating primary microcephaly were investigated. Families were identified through public networks and visited at their homes. After informed consent, multiple elders were interviewed for family history of the disease. Peripheral blood samples were collected from affected as well as healthy individuals in EDTA coated vacutainers and stored at 4ºC.

### Genetic Analysis:

***Whole Exome sequencing:*** Genomic DNA was extracted from peripheral blood samples. For genetic analysis, Whole Exome Sequencing (WES) of probands IV:2 from family A and IV:1 from family B was performed using Agilent (Santa Clara, CA) version 6 enrichment kit and the Illumina HiSeq 4000 sequencing system (paired-end reads, 2x75by). The experiments and next-generation sequencing data handling were conducted as reported by Moawia et al.^11^ The sequence reads were aligned with GRCh37 human genome assembly. The variants were filtered and prioritized using an in-house VARBANK database and analysis tool kit.^11^ Minor allele frequency (MAF) <1.5% was set as a cut-off value for variants (single nucleotide variants/small indels) in the coding region and the flanking intronic regions (68bp). The MAF cut-off value for known causal variants (Human Genetic Variation database) was up to 5% and up to ± 30 bp of flanking regions. MAFs were taken from the following databases: dbSNP, 1000 Genomes, gnomAD, and an in-house database. The coverage value was 30 high-quality sequencing reads per base for more than 98% of the targeted regions. Considering the consanguinity of the parents and autosomal recessive inheritance, only homozygous variants were selected.

### Sanger Sequencing:

Bi-directional Sanger sequencing was performed for co-segregation analysis of variants in both families. Target regions were amplified using primers sets ASPM_Ex26_F (TTGGTTGGGTTGTTTGTAAATG), ASPM_Ex26_R (TTTATCCGTGCAAAAAGCAG), CDK5RAP2_Ex6_F (5’ TGCATGTTTTACCCCTGTGC 3’) and CDK5RAP2-Ex6_R (5’ TGCCAGCCTATTATTAACCCAC 3’). The amplification products were cleaned by treating with Exonuclease I and Shrimp Alkaline Phosphatase. Amplified PCR products were sequenced using Big Dye Terminator v.3.1 Cycle Sequencing Kit (Applied Biosystems, CA, USA). Sequencing PCR products were resolved in an ABI3130xl genetic analyser (Applied Biosystems, CA, USA). Sequence files were analyzed using ChromasPro 1.7.6 software.

## RESULTS

### Clinical features:

**Fig.1:**
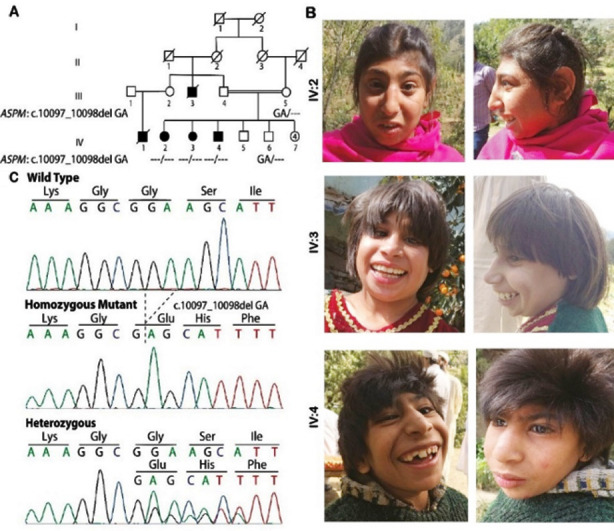
(A) Pedigree of family A showing consanguinity of parents, autosomal recessive inheritance of disease, and genotype of each individual analyzed. (B) Clinical features of the affected individuals showing reduced head circumference and sloping forehead. (C) Sequence chromatogram of part of *ASPM*, exon 26 (NM_018136.5) obtained from a healthy control (top), affected individual IV:2 (middle), and healthy sibling IV:5 (bottom).

Family A originated from District Swat of Pakistan and is comprised of nine siblings born to consanguineous parents (Fig.1A). Two females (IV:2, IV:3) and one male (IV:4) manifested reduced HC (SD -10 to -12) with sloping forehead (Fig.1B), ID, reduced height along with seizures (IV:2 and IV:5), and ocular, speech, and salivation abnormalities as detailed in Table-I.

**Table I:** Clinical features of affected individuals from family A and family B.

Family ID	Individual ID	Gender	Age (Y)	HC (cm)	HC (SD)	Height (cm)	Height (SD)	Intellectual Disability	Loss of Self-care	Speech Impairment	Hearing Impairment	Ocular Anomalies	Ptyalism	Seizures	Aggressive	Hyperactive
Family A	IV:2	F	12	41	-10	122	-3	++	++	+++	-	Strabismus	+	++	-	++
	IV:3	F	9	38	-12	99	-5	++	+++	+++	-	Strabismus	+	-	-	+
	IV:4	M	6	36	-11	96	-5	++	+++	+++	-	Strabismus	+	++	-	+++
Family B	IV:1	M	30	43.5	-8	155	-3	+	+	+++	+++	-	-	+	+	-
	IV:2	F	32	44	-8	147	-3	+	+	+++	+++	-	-	+++	+++	-

F= Female, M= Male, S.D = Standard Deviation, HC= Head Circumference, Y= Years, Absent = -, Mild = +, Moderate = ++, Severe = +++

**Fig.2:**
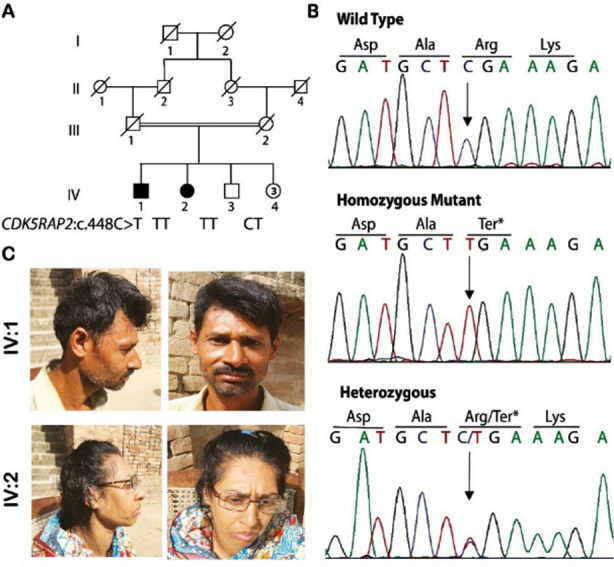
(A) Pedigree of family B showing consanguinity of parents, autosomal recessive inheritance, and genotypes of each individual for the variant *CDK5RAP2*: c.488C>T (B) Frontal and side view of the patients showing microcephaly and sloping forehead with otherwise normal facial features. (C) Sanger traces of part of *CDK5RAP2* exon 6 (NM_018249.5) obtained from a healthy control (top), affected individual IV:1 (middle), and healthy sibling IV:3 (bottom). The arrow indicates the position of the mutation.

Family B was ascertained from district Hafizabad, Punjab of Pakistan, and has six siblings born to consanguineous parents (Fig.2A). One male (IV:1) and one female (IV:2) showed congenital microcephaly (SD -10 and -9 respectively), with sloping forehead (Fig.2B), ID, seizures and speech, and hearing impairments, as summarized in Table-I. No signs of developmental delay, growth retardation, skeletal anomalies, and visual impairment were observed.

### Genetic findings:

WES data of family A revealed a novel frameshift mutation (NM_018136.5; c.10097_10098delGA) in exon 26 of the *ASPM* gene, substituting Glycine to Glutamic acid at 3366 amino acids, and causing premature truncation. Polyphen-2 predicted the variant to be “probably damaging” (score 0.917). Mutation taster strengthened the prediction of Polyphen-2 by predicting it as “disease-causing” through frameshift, change in amino acid sequence, splice site changes, and non-sense mediated decay (NMD). PhastCons score of this deletion ranged between 0.997-1 (0; No conservation, 1; high conservation of the element in 46 species. It considers the flanking columns of the variation).

WES data analysis of family B identified a previously reported homozygous variant (NM_018249.5; c.448C>T; p.(Arg150*)) in *CDK5RAP2* gene.^4^ It was a nonsense mutation in exon 6 of *CDK5RAP2*, predicted to result in a premature termination codon. Bidirectional Sanger sequencing of the whole family confirmed the co-segregation of the variant with the disease (Fig.2 A & C). Variant(s) in any other MCPH gene have not been detected in both families

## DISCUSSION

This study reports genetic and clinical features for primary microcephaly in two families. MCPH is reported to be caused by biallelic mutations in at least 28 different genes. There is a little genotype to phenotype correlation and patients are clinically indistinguishable.^4^ Considering this genetic heterogeneity, WES was adopted to delineate causal variants. WES covered 99% protein-coding part of the genome, where 85% of known disease-causing variants for Mendelian disorders reside.^12^ In addition to screening all known MCPH genes at once, it also offers the possibility of identification of mutations in novel genes.^11^ Classical techniques like Sanger sequencing/ gene panel sequencing have been beneficial but are more time consuming, less economic, and laborious.^13^,^14^

### The mutation ASPM:

c.10097_10098delGA, p.(Gly3366Glufs*19) identified in family A is predicted to alter the open reading frame, introducing 19 unrelated residues before introducing a premature termination codon. The mutation resides close to the C-terminus of the protein that lacks any known domain. Nevertheless, loss of function mutations in these regions have been reported to be pathogenic.^4^,^15^ Since it is a truncating mutation, the mutant transcript is highly likely to undergo non-sense mediated decay, leading to the absence of protein. The variant is not catalogued in databases of human polymorphism like gnomAD, 1000 genomes, dbSNP150, and an in-house database at Cologne Centre for Genomics with >1,600 exomes. So far, 211 mutations have been reported in this gene that are evenly distributed across the 28 exons of this gene.^4^ The current study increases the number to 212, thus adding to the mutational spectrum of the gene. *ASPM* mutations do not show great clinical heterogeneity or strong genotype-phenotype correlation.^16^,^17^ The clinical symptoms of our patients are in line with previous reports of *ASPM* related microcephaly presenting moderate ID, speech impairment, visual abnormalities, seizures, and ptyalism.^4^,^15^,^18^,^19^

### The mutation CDK5RAP2:

c.448C>T p. (Arg150*) segregating in family B is predicted to cause very early termination of the translation. The mutant transcript is predicted to undergo NMD and thus produce no protein. The mutant protein, if made in case of incomplete NMD, would be missing functionally critical domains thus would be non-functional. Loss of this protein is known to impede microtubule nucleation and affect cell cycle dynamics leading to premature neuronal differentiation and early depletion of neuro-progenitor cells causing MCPH and allelic disorder Seckel syndrome.^20^-^22^

### The mutation CDK5RAP2:

c.448C>T p. (Arg150*) is catalogued in gnomAD with only 4 allele counts that are all heterozygous. It is also present in dbSNP with an identifier of rs771565845 and allele count of two and it is absent in ClinVar. The variant has previously been reported in only one family, where patients presented with delayed developmental milestones, mild to moderate ID, speech impairment, standard self-care, and motor skills.^4^ Our patients presented similar features except for developmental delay. There are some reports of short stature in patients with a mutation in this gene^4^,^23^ that is also observed in our cases thus it provides additional evidence for its role in the determination of height too. Interestingly, our patients also showed a history ofmild to severe seizures without ptyalism that is rarely reported feature of patients with mutations in *CD5RAP2*.

### Limitations of the Study:

The genomic analyses provide very convincing evidence to the pathogenicity of the identified variants; the study however has the limitation that their functional validation has not been performed and thus their precise pathomechanism cannot be envisaged.

## CONCLUSION

Conclusively, we report a novel mutation in *ASPM*, increasing the mutation spectrum to 212. We also report an ultra-rare mutation in *CDK5RAP2*, adding supporting evidence to the pathogenicity of this variant. It also identified atypical features in patients having *CDK5RAP2* mutation, thus expanding the clinical spectrum of MCPH3 associated microcephaly. The study demonstrates the efficacy of whole-exome sequencing to delineate the genetic etiology of heterogenous disorders like primary microcephaly. Together these findings will contribute towards improved diagnostic and preventive strategies to control the incidence of this disorder.
